# Progress in Neurology

**Published:** 1901-04-20

**Authors:** 


### Progress in Neurology.

**Electrical Treatment of Hemiplegia and Sciatica.**—Lewis Jones[1] says that though it is useless to expect any result from electrical treatment of cases of hemiplegia with marked secondary rigidity, yet in recovering cases their progress can be much hastened. This is due to the fact that round the area of actual destruction in the brain there is a damaged though recovering portion. It is found in such cases that brisk peripheral stimulation of the affected limbs by the induction coil is followed by a marked gain of power. The greater part of the good that may be done by electricity occurs within two months of the time of beginning treatment; at the end of that time the cases have gained all that is possible. In the treatment of sciatica the constant current should be used, with the positive pole in contact with the painful part. The negative pole should be placed on the abdomen in the iliac region, and the positive moved up and down over the line of the sciatic nerve, and in those parts especially where the patient feels most pain. The secret of success is to use large electrodes—about 3 inches in diameter—and a strong current. The skin may be slightly soaped so that the electrode may glide smoothly. Though cases of old standing are very tedious, yet recent ones are rapidly relieved. Rest, warmth and electricity are the essentials in the treatment of sciatica.

**Ocular Headaches.**—According to Hinshelwood[2] at least 50 per cent. of cases of chronic headaches are due to eye-strain. Many of these cases are not recognised because the connection between the headache and the eyes is not always manifest. The headache is usually a dull pain or a feeling of weight and heaviness, or exceptionally it may be intense. It may be frontal, temporal, vertical, or occipital; but it is commonest in the frontal region just above the orbits. In many cases this headache is accompanied by discomfort in or about the eye—such as a sense of heaviness or burning in the lids, soreness in the globe of the eye, or pain at the back of the orbit; in these cases the cause is readily suspected. But there are many cases where there is no discomfort about the eyes, and in them the cause of the headache is readily overlooked. Ocular headache is generally brought on or intensified by the use of the eyes, or relieved or ameliorated by resting them. Exceptionally, though the eye-strain is continuous, the headaches are paroxysmal, somewhat resembling megrim, though bilateral in distribution. Headaches of ocular origin are frequently due to very slight errors of refraction; high errors are rarely associated with headache—this is probably due to the patient constantly trying to correct the error in the former case. It is only in about 25 per cent. of cases of refractive error that headache occurs; and of all forms of refractive error the one which is most frequently the cause of headache is astigmatism. Thus of 123 cases of refractive error associated with headache, 90 were cases of astigmatism, 28 cases of hypermetropia, and 5 cases of myopia. Of the 90 cases, 40 were cases of hypermetropic, 31 cases of myopic, and 19 were cases of mixed astigmatism. A very small amount of astigmatism, 0·5 to 1 D. is very frequently the cause of the headache; these errors cause an excessive strain to be thrown on the ciliary muscle. Another cause, though a much rarer one, of ocular headache is weakness of the external muscles of the eyes, most commonly of the internal recti which are used for long hours together in keeping the eyes in a state of convergence. This should be kept in mind when correction of refractive error does not give relief. Suitable glasses will in many cases not only correct the error of refraction but also the headache. In others, however, additional medical treatment is necessary to bring about the desired result. These are cases where the headache is the result of several contributing factors, of which refractive error is only one. Thus the patient may have just passed through some debilitating illness, or be anæmic, or run down by long continued work.

**Internal Hydrocephalus following Cerebro-spinal Meningitis.**—Joslin[3] has recorded eight cases of this condition, given a *résumé* of the literature on the subject, and come to the following conclusions. The hydrocephalic fluid is clear and colourless, and sterile. The pia-mater is thickened in the region of the roof of the fourth ventricle, and in most cases elsewhere as well, particularly at the base of the brain. The thickening renders the whole roof of the fourth ventricle impervious and closes the openings in it. As a result a condition of hydrocephalus develops, the flattening of the convolutions varying with the amount of fluid. The ependyma may be normal, or velvety, or thickened. Clinically, it is found that either the active stage of the meningitis merges into a condition which is directly attributable to the hydrocephalus, or there is an interval of apparent convalescence after the meningeal symptoms have subsided. No relation exists between the development of the hydrocephalus and the severity of the original attack. It is not so much the extent and intensity of the initial inflammation as its location which is of importance. Apathy or mental lassitude is the predominant factor in the symptomatology; this increases until the patient appears as if asleep the greater part of the time. He can be easily roused, but only to drop off again into his former condition. This case may pass into one of unconsciousness or somnolence. Irregular vomiting with no relation to diet is very frequent. Headache is severe in one-half of the cases; in general it is far less severe than the headache of onset. The pupils are dilated or react sluggishly. Muscular weakness and emaciation are usual, but not out of proportion to the length of the disease and the small diet. The temperature is normal or subnormal. A striking feature of these cases is their long duration—from 50 to 130 days. Thus mental apathy, headache, vomiting, dilated or sluggish pupils, with a normal or subnormal temperature, when they occur a month or more after the onset of cerebro-spinal meningitis, point to hydrocephalus. Prognosis is generally very bad, though exceptionally it would seem complete or incomplete recovery takes place. No treatment is of any avail. Tapping the lateral ventricles and lumbar puncture have been tried, but are useless.

**Pathology of Herpes Zoster.**—Head and Campbell[4] have made an extensive series of observations on the pathology of herpes zoster, founded on cases where the patient died at various periods subsequently. The acute changes found in the posterior root ganglion of the nerve supplying the affected area of the skin, consist of (1) an extensive acute inflammation with the exudation of small round deeply-staining cells; (2) extravasation of blood; (3) destruction of ganglion cells and fibres; (4) inflammation of the sheath of the ganglion. If severe such a condition leaves a scar in that part of the ganglion affected and leads to thickening of the sheath above the affected area. On the other hand if the eruption has not been severe, all traces of the inflammation present in the acute stage may pass away leaving the ganglion apparently normal. The changes in the posterior nerve root corresponded to the results which might have been expected from the lesion of the ganglion; they consisted of an acute degeneration followed by a greater or less amount of secondary sclerosis according to the severity of the acute destruction. The anterior root was in all cases normal. In the mixed peripheral nerves degeneration likewise occurs, and can be traced right up to the fine twigs which pass into the skin to supply the area over which the eruption is distributed. The time relations of the degeneration and subsequent sclerosis to the eruption was the same in the peripheral nerve and the posterior root. The degeneration in the posterior roots can be traced into the posterior columns of the spinal cord. Where the eruption extends on to the arm the degenerated fibres can be followed in the cord from the root zone to the postero-external column, and by this path up to the nucleus cuneatus. Where the eruption is on the leg the field of degeneration passes into the postero-median column. The posterior roots in the dorsal region, containing as they do afferent fibres from the trunk, consist mainly of short fibres which do not form a part of either the postero-internal or the postero-external columns in the cervical region of the spinal cord, but run up in the root zone adjacent to the posterior horn, get less in number as they ascend and disappear in a variable (6 to 16) number of segments. Thus the long fibres which form the postero-internal and postero-external columns in the cervical region come almost exclusively from the leg and arm respectively. Zoster of the branches of the trigeminal is associated with a similar lesion in the Gasserian gauglion to that found in the posterior root ganglia in cases of zoster of the trunk and limbs. This lesion causes secondary degeneration in the sensory root of the Gasserian ganglion both in its extra- and intra-medullary course. Zoster in all respects resembling that arising spontaneously may be produced by implication of a posterior root ganglion in inflammatory processes secondary to malignant disease, tubercle, or injury.

Sections through an unbroken vesicle of herpes zoster show a cavity, the floor of which is formed of naked papillæ which are in a condition of profound inflammation and are infiltrated with small round cells which stain deeply. The cavity of the vesicle is filled with fluid which contains broken-down epithelial cells of all sizes and shapes and small round cells. No sign of micro-organisms either in the vesicle or its surroundings can be found. The lymphatic glands enlarge and frequently become tender, and are found on section to be in a condition of inflammation and free from micro-organisms. Thus there is the curious fact that a collection of inflamed vesicles containing a sterile fluid gives rise to enlargement and inflammation of the lymphatic glands which also show no sign of bacterial invasion.

Herpes Zoster must be considered an acute specific disease of the nervous system, in which the febrile period lasts from three to five days. The rash may appear a few hours after the onset of the disease, or may tarry till the fall of temperature. As in the case of other specific diseases second attacks are very uncommon. An exactly analogous disease is acute anterior poliomyelitis, which similarly begins with malaise and fever lasting from three days to a week, and at a variable time during this febrile period paralysis is noted. Again, the pathological anatomy of acute anterior poliomyelitis is exactly similar to that of the posterior root ganglion in herpes zoster. Herpes zoster might justly be spoken of as acute posterior poliomyelitis. The changes in the posterior root ganglion consist of an acute interstitial inflammation accompanied by necrosis of the ganglion cells. Of the nature of the agent which is responsible for this we are completely ignorant. This agent commonly attacks one ganglion only. The ganglion most commonly affected are those which receive afferent impulses from the viscera through the white ramus of the sympathetic, and which (anatomically) contain a preponderance of the smaller type of ganglion cells that give rise to the shorter fibres of the posterior columns. These smaller cells among other functions probably subserve those of pain, for the long tracts of the posterior columns do not conduct pain impressions. Hence the intense pain which accompanies an attack of zoster. The eruption is probably produced not by disturbance of special trophic nerves but by irritation of cells in the ganglion which subserve the function of pain, and more particularly that form of pain produced by afferent visceral impulses.

[1] Lewis Jones, Clin. Jour., Dec. 5, 1900. [2] Henshelwood, Glasgow Med. Jour., Nov. 1900. [3] Joslin, Amer. Jour. Med. Sci., Oct. 1900. [4] Head and Campbell, Brain, Autumn Number, 1900.

